# Spectral dynamic causal modeling: A didactic introduction and its relationship with functional connectivity

**DOI:** 10.1162/netn_a_00348

**Published:** 2024-04-01

**Authors:** Leonardo Novelli, Karl Friston, Adeel Razi

**Affiliations:** Turner Institute for Brain and Mental Health, School of Psychological Sciences, and Monash Biomedical Imaging, Monash University, Australia; Wellcome Centre for Human Neuroimaging, University College London, London, United Kingdom; CIFAR Azrieli Global Scholars Program, Toronto, Canada

**Keywords:** Effective connectivity, Functional connectivity, State-space modeling, fMRI

## Abstract

We present a didactic introduction to spectral dynamic causal modeling (DCM), a Bayesian state-space modeling approach used to infer effective connectivity from noninvasive neuroimaging data. Spectral DCM is currently the most widely applied DCM variant for resting-state functional MRI analysis. Our aim is to explain its technical foundations to an audience with limited expertise in state-space modeling and spectral data analysis. Particular attention will be paid to cross-spectral density, which is the most distinctive feature of spectral DCM and is closely related to functional connectivity, as measured by (zero-lag) Pearson correlations. In fact, the model parameters estimated by spectral DCM are those that best reproduce the cross-correlations between all measurements—at all time lags—including the zero-lag correlations that are usually interpreted as functional connectivity. We derive the functional connectivity matrix from the model equations and show how changing a single effective connectivity parameter can affect all pairwise correlations. To complicate matters, the pairs of brain regions showing the largest changes in functional connectivity do not necessarily coincide with those presenting the largest changes in effective connectivity. We discuss the implications and conclude with a comprehensive summary of the assumptions and limitations of spectral DCM.

## INTRODUCTION

Dynamic causal modeling (DCM) refers to the Bayesian fitting of state-space models to explain observed physiological signals in terms of hidden neuronal activity and connectivity (K. Friston, Harrison, & Penny, 2003; Triantafyllopoulos, 2021). The distinction between observed and hidden variables is particularly relevant in neuroscience because the signals recorded via noninvasive neuroimaging are not a direct measurement of neuronal states or connectivity. In fact, using observed recordings to infer unobserved neural interactions is the main purpose of DCM and the reason for its widespread adoption. It is also a distinctive feature that sets it apart from functional connectivity analysis, which simply characterizes statistical dependencies in observed time series. Over the last 20 years, the versatility offered by state-space models has seen DCM applications in most neuroimaging modalities (Frässle et al., 2021; K. Friston et al., 2003, 2019; K. J. Friston, Kahan, Biswal, & Razi, 2014; Jung, Kang, Chung, & Park, 2019; Kiebel, Garrido, Moran, & Friston, 2008; Moran et al., 2009; Tak, Kempny, Friston, Leff, & Penny, 2015), along with recent applications to epidemiology (K. J. Friston, Flandin, & Razi, 2022) and beyond (Bach, Daunizeau, Friston, & Dolan, 2010). Navigating the vast and technical DCM literature, however, is by no means a trivial task—especially to the novice learner. Happily, there are excellent introductory resources on individual- and group-level analysis using deterministic versions of DCM, which are designed for neuroimaging experiments involving behavioral tasks (Stephan, 2004; Stephan et al., 2010; Zeidman, Jafarian, Corbin, et al., 2019; Zeidman, Jafarian, Seghier, et al., 2019). A recent primer on variational Laplace explains how Bayesian inference is performed in DCM (Zeidman, Friston, & Parr, 2023) using the SPM software (https://www.fil.ion.ucl.ac.uk/spm). However, there is a lack of introductory material on DCM for resting-state data analysis, despite the remarkable growth of the resting-state paradigm and the widespread uptake of these methods. Here, we fill this gap with a didactic introduction to spectral DCM that aims to explain its technical aspects (K. J. Friston et al., 2014; Razi, Kahan, Rees, & Friston, 2015).

**Figure 1.**
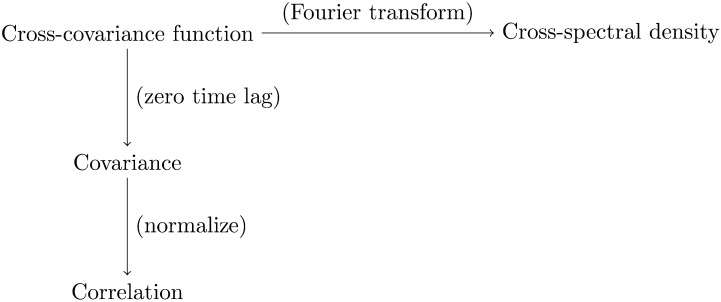
The distinctive feature of spectral dynamic causal modeling (DCM) is the focus on modeling the measured cross-spectral density (top right), which is a second-order summary statistic of the time series data. This is closely related to Pearson’s correlation (bottom left), another second-order statistic and the most widely used measure of functional connectivity in neuroimaging. In fact, both can be derived from the cross-covariance function (top left). The cross-spectral density is obtained directly via the Fourier transform (horizontal arrow). As such, it is the equivalent representation of the cross-covariance function in the frequency domain instead of the time domain. The correlation is obtained in two steps (vertical arrows). First, we compute the covariance as a special case of the cross-covariance function between two time series, by setting the time lag between them to zero. Second, we normalize the covariance such that its values are restricted to the [−1, 1] interval to obtain the zero-lag correlation. For the mathematical relationships among these quantities, we refer readers to K. J. Friston et al. (2014, Figure 1).

What distinguishes spectral DCM from other DCM versions, and when should we choose it? Firstly, spectral DCM employs random differential equations instead of deterministic ones. These are used to model spontaneous endogenous fluctuations in neuronal activity, enabling resting-state analysis in the absence of experimental inputs. But its distinctive feature is the focus on modeling the measured cross-spectral density, which is a second-order summary statistic of the time series data. This is closely related to Pearson’s correlation, another second-order statistic and the most widely used measure of functional connectivity in neuroimaging (see diagram in Figure 1; for mathematical relationships, also see K. J. Friston et al., 2014, Figure 1). In fact, the correlation is obtained by normalizing the covariance such that its values are restricted to the [−1, 1] interval. In turn, the covariance is a special case of the cross-covariance function between two time series, when there is no time lag between them. Finally, the Fourier transform of the cross-covariance function gives the cross-spectral density (under stationarity assumptions). In other words, the cross-spectral density is the equivalent representation of the cross-covariance function in the frequency domain instead of the time domain—an important relationship that we will unpack later in this article.

Spectral DCM fits the parameters of a linear, continuous-time model to the observed cross-spectral density. The estimated parameters are those that best reproduce the cross-correlations between all variables, at all time lags. In particular, the estimated effective connectivity also reproduces the zero-lag correlations between the observed time series—the most common measure of functional connectivity in the literature. This would be appealing to researchers who are interested in both effective and functional connectivity. The nuanced relationship between effective and functional connectivity is explored in the Effective and Functional Connectivity section. Prior to that, we introduce and explain the various components of the generative model, that is, the model that generates the cross-spectral density given a set of parameters. These basic building blocks are used routinely in signal processing and control theory and are often presented only briefly in the DCM literature. Here, we adopt an inclusive and slower pace for those who are not familiar with state-space models and spectral data analysis. That said, we count on the reader to fill in the gaps and look up concepts such as the Fourier transform (Oppenheim, Willsky, & Nawab, 1997; Smith, 2002) or convolution (Smith, 2002), if needed. Moving from theory to practice, a step-by-step guide to running spectral DCM on a real resting-state functional MRI (fMRI) dataset is provided in Chapter 38 of the SPM12 manual (Ashburner et al., 2020).

A final reason for choosing spectral DCM is its computational advantage compared with stochastic DCM (Li et al., 2011). It is important to note that the lower computational complexity and the resulting increase in speed rely on the assumption that the statistics of endogenous neuronal fluctuations are conserved over the experimental time window, making spectral DCM suitable for resting-state neuroimaging experiments. Experimental inputs can also be included via an additional term in the model, although applications to task experiments are infrequent in the literature. Introducing even stronger assumptions leads to even faster schemes, such as *regression DCM*, which can analyze hundreds of brain regions in minutes (Frässle et al., 2021). However, this method forgoes the strict separation between hidden and observed variables that is typical of state-space modeling and that we have used to define DCM herein. As the name suggests, regression DCM is more akin to the Bayesian fitting of a multivariate autoregressive model in the frequency domain.

The key assumptions made in spectral DCM are summarized in the Assumptions and Limitations section.

## BUILDING THE GENERATIVE MODEL, ONE ELEMENT AT A TIME

The signals recorded via noninvasive neuroimaging are not a direct measurement of neuronal activity. In the case of fMRI, the observed blood-oxygen-level-dependent (BOLD) signal captures changes in blood oxygenation that indirectly reflect neuronal activity. For this simple reason, spectral DCM models the neuronal and the observed variables separately (denoted by *x* and *y*, respectively). Such a distinction represents both the main strength and the challenge of the DCM framework.

### Neuronal Model

In spectral DCM, the neuronal model is defined by the linear random differential equation

$$\dot{x}\left(t\right)=Ax\left(t\right)+v\left(t\right),$$ (1)

where ***x***(*t*) is the *n*-dimensional *state vector*

$$x\left(t\right)=\left[\begin{array}{c} x_{1}\left(t\right)\\ \vdots \\ x_{N}\left(t\right) \end{array}\right]$$

whose *N* scalar components represent different brain regions. These are called *state variables* in the state-space modeling literature (Durbin & Koopman, 2012; Williams & Lawrence, 2007). The time derivative of the state vector is denoted as $\dot{x}$ (*t*), where differentiation with respect to time is performed component-wise, that is, $\dot{x}\left(t\right)={\left[{\dot{x}}_{1}\left(t\right),\ldots ,{\dot{x}}_{N}\left(t\right)\right]}^{⊺}$. The activity of the system is sustained by stochastic, non-Markovian, endogenous fluctuations denoted as ***v***(*t*), which we will consider in the Endogenous Fluctuations section. Let us first turn our attention to the *A* matrix, which defines and parameterizes the *effective connectivity*.

### Effective Connectivity

The effective connectivity quantifies the directed effect of one brain region on another. Effective connectivityThe directed effect of one brain region on another, measured as a rate of change. To better understand its meaning, consider a simple deterministic system with two brain regions and no stochastic components. The matrix-vector notation in Equation 1 can be unpacked into two scalar components,

$${\dot{x}}_{1}\left(t\right)=a_{11}x_{1}\left(t\right)+a_{12}x_{2}\left(t\right),$$ (2a)

$${\dot{x}}_{2}\left(t\right)=a_{21}x_{1}\left(t\right)+a_{22}x_{2}\left(t\right),$$ (2b)

where *a*_*jk*_ corresponds to the element in the *j*th row and *k*th column of *A*. More explicitly, in this example, we have

$$A=\left[\begin{array}{cc} a_{11} & a_{12}\\ a_{21} & a_{22} \end{array}\right].$$ (3)

Let’s initially assume that *x*_1_ is inactive at time *t*_1_ and set *x*_1_(*t*_1_) = 0 in Equation 2a. We get

$${\dot{x}}_{1}\left(t_{1}\right)=a_{12}x_{2}\left(t_{1}\right),$$ (4)

stating that the instantaneous change in *x*_1_ is proportional to the input from *x*_2_. The effective connectivity *a*_12_ is simply the coefficient that determines the *rate* of such change. Therefore, in DCM, the effective connectivity *a*_*jk*_ quantifies the instantaneous response rate of *x*_*j*_ caused by a change in *x*_*k*_, in the ideal case where all other variables were kept fixed or set to zero (readers who are familiar with multivariate calculus would recognize this as a partial derivative and the *A* matrix as a Jacobian). Being a rate, effective connectivity is always measured in hertz (change per second).

**Figure 2.**
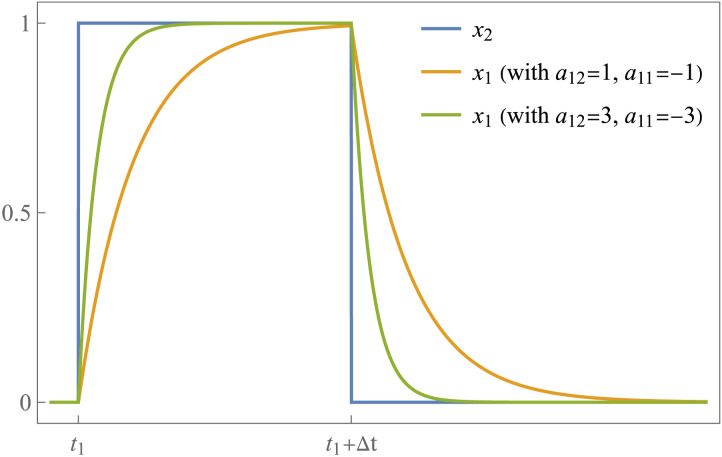
The role of the effective connectivity parameters in a deterministic linear system with two variables. The parameter *a*_12_ determines the instantaneous rate of response of *x*_1_ to an input from *x*_2_. In this example, the input is constant with duration Δ*t*. The initial slope of the response is steeper for higher values of *a*_12_. Once the input from *x*_2_ ceases, the activity of *x*_1_ decays exponentially in the absence of other inputs. The decay rate is determined by the self-connection *a*_11_, with larger negative values resulting in faster decay (shorter memory).

Figure 2 shows the impact of *a*_12_ on the response of *x*_1_ to a constant input from *x*_2_ with duration Δ*t*. Note that the effective connectivity determines the initial slope of the curve, which is steeper for high values of *a*_12_. However, once the input from *x*_2_ ceases, the magnitude and duration of the response in *x*_1_ no longer depend on *a*_12_; instead, they only depend on the self-connection *a*_11_. That is, after the time interval Δ*t*, we have *x*_2_(*t*) = 0 and Equation 2a becomes

$${\dot{x}}_{1}\left(t\right)=a_{11}x_{1}\left(t\right),$$ (5)

which has the simple exponential solution

$$x_{1}\left(t\right)={ce}^{a_{11}t},$$ (6)

for each time *t* > (*t*_1_ + Δ*t*), where the constant factor *c* is the value of *x*_1_ when the input from *x*_2_ ceases, that is, *c* =*x*_1_(*t*_1_ + Δ*t*). It is useful, and biologically plausible, to impose a negativity constraint on the rate constant of the self-connections (i.e., *a*_11_) to avoid instability and divergence to infinity. In this example, a negative value of *a*_11_ guarantees that *x*_1_(*t*) converge to zero. In the multivariate case, the stability of a linear dynamical system is guaranteed when all the eigenvalues of the effective connectivity matrix *A* have negative real parts (Izhikevich, 2006).

(Note: The reason why DCM studies often report positive values on the diagonal of the effective connectivity matrix *A* is that the self-connections are transformed using the logarithmic function log(−2*a*) by SPM. This convention is technically motivated by the use of log-normal priors to enforce positivity or negativity on certain parameters; here, to enforce recurrent or self inhibition. A reported zero value for a self-connection corresponds to −0.5 Hz, which is the default prior self-connectivity value in SPM12. Negative values correspond to slower decay rates in the (−0.5, 0) range, while positive values correspond to faster decays, that is, <−0.5 Hz.)

### Power Spectral Density and Cross-Spectral Density

A time-varying signal *z*(*t*) is a function of time. However, the same function could be represented as a sum of elementary sine waves, each characterized by a single frequency. This sum is weighted, with some frequencies carrying more weight than others (i.e., the sine waves can have different amplitudes). Every function, *z*(*t*), is a unique mix of frequencies, some more pronounced, some less. This unique profile is called Fourier frequency spectrum. The time- and frequency-domain representations of a function are two sides of the same coin: They are equally informative but reveal different and complementary aspects of the same data. The Fourier transform (𝓕) is the mathematical tool that turns the coin over: It converts a function of time into the corresponding function of frequency (while the inverse Fourier transform does the opposite). If we denote the (angular) frequency by *ω*, then 𝓕 turns the time function *z*(*t*) into the frequency function *Z*(*ω*). Mathematically, this transformation is achieved via the integral

$$Z\left(\omega \right)=𝓕\left\{z\left(t\right)\right\}=\int_{-\infty }^{\infty }z\left(t\right)e^{-i\omega t}\text{d}t$$ (7)

(for time signals, we often only consider positive values of *t* and compute the integral in the [0, ∞] interval; this is equivalent to setting *z*(*t*) = 0 for all *t* < 0). The resulting *Z*(*ω*) is a function of the frequency *ω* and no longer depends on time. Somewhat ambiguously, the term “Fourier transform” is used to denote both the mathematical operation and the resulting function, *Z*(*ω*). Note that *Z*(*ω*) typically returns complex values, owing to the presence of the imaginary unit *i* in Equation 7. Yet, the squared magnitude of a complex number (e.g., |*Z*(*ω*)|^2^) is a real number, defined as the square of its real part plus the square of its complex part. Therefore, the magnitude of the Fourier transform is a function that returns only real values, which makes it easier to understand and visualize. This function is the *power spectral density* of the signal. The simplicity of interpretation comes with a loss of information. After computing the squared magnitude to obtain the power spectral density, we cannot go back and recover the original complex-valued Fourier transform (similarly to what happens for real numbers, where the square produces a unique result but the square root has two solutions). A similar information loss affects all second-order properties of the time series, including the cross-spectral density and the (cross-) correlation, which are two key concepts that we will discuss and connect later.

Until now, we have considered only deterministic signals. However, spectral DCM is concerned with stochastic (nondeterministic) processes, such as the endogenous fluctuations that we will examine in the next section, which are a proxy for thoughts or mind-wandering-like processes during resting-state brain activity. A stochastic process is a sequence of random variables. If *x* is a stochastic process indexed by time, then *x*(*t*) is not a number but a random variable with a given probability distribution (see Figure S1 in the Supporting Information for an illustration). The simplest example is the white noise process, which follows a normal distribution at each time point, independent from previous time points. The rest of this paragraph explains why white noise has a flat power spectral density and is meant for the mathematically versed reader. According to stochastic calculus, the Fourier transform of a stochastic process is also a stochastic process; however, it is indexed by frequency instead of time. In the case of white noise, each frequency *ω* corresponds to a distinct random variable that follows the same complex normal distribution as the other frequencies but is independent of them. In turn, the power spectral density is also a stochastic process indexed by frequency, obtained as the squared magnitude of the Fourier transform. Therefore, in the case of stochastic processes, we will consider the expected value of the power spectral density (𝔼[|*Z*(*ω*)|^2^]), which is a number, that is, a scalar function of frequency. Being the expectation of the squared magnitude, the power spectral density can also be understood as the variance of the Fourier transform of a stochastic process, if the latter has zero mean:

$$\text{Var}[𝓕\left\{z\left(t\right)\right\}]=\text{Var}\left[Z\left(\omega \right)\right]=𝔼\left[{\left|Z\left(\omega \right)\right|}^{2}\right]+{\left|\underset{=0}{\underbrace{𝔼\left[Z\left(\omega \right)\right]}}\right|}^{2}=𝔼\left[{\left|Z\left(\omega \right)\right|}^{2}\right].$$ (8)

In conclusion, white noise has a flat power spectral density because the variance of its Fourier transform is the same for all frequencies. (Technical note: for simplicity, we assume that the Fourier transform exists. The general definition of the power spectral density involves a limit over the bounds of the Fourier integral; Miller & Childers, 2012).

From here, we can seamlessly transition to multivariate stochastic processes using the same mathematical tools. If ***x***(*t*) is a vector with one element per brain region, its Fourier transform ***X***(*ω*) is also a vector. This is important because it applies to the stochastic neuronal variable in Equation 1. The multivariate analogue of the power spectral density is the *cross-spectral density*, defined as the covariance matrix

$$G_{x}\left(\omega \right)=\mathrm{cov}\left[X\left(\omega \right),X\left(\omega \right)\right]=𝔼\left[X\left(\omega \right)X{\left(\omega \right)}^{†}\right],$$ (9)

where † indicates the conjugate transpose of the vector ***X***(*ω*). The dot product between the column vector ***X***(*ω*) and its conjugate transpose is a square matrix. Specifically, *G*_*x*_(*ω*) is an *N* × *N* matrix whose diagonal elements are the power spectral densities (variances) of individual neuronal variables, representing various brain regions. These are real positive numbers. Each off-diagonal element describes the cross-spectral density (covariance) between a different pair of variables. Unlike the diagonal elements, they generally take complex values.

Admittedly, the cross-spectral density definition as a covariance in the frequency domain is quite abstract. A better intuition will develop after exploring the close relationship between cross-spectral density and functional connectivity. In the Cross-Spectral Density and Functional Connectivity section, we’ll see how the cross-spectral density is the Fourier transform of the cross-covariance function, which captures both the correlation matrix and its time-lagged extensions. For now, the power spectral density definition given above is sufficient to understand how endogenous fluctuations are modeled in spectral DCM.

### Endogenous Fluctuations

Stable deterministic linear systems can only converge to a state of permanent equilibrium or produce an infinite sequence of identical oscillations. To overcome these limitations and add variability to the neuronal oscillations, we can introduce endogenous (intrinsic) fluctuations in the system, sometimes referred to as *state noise*. Endogenous fluctuationsIntrinsic stochastic fluctuations that serve as a proxy for thoughts or mind-wandering-like processes during resting-state brain activity. For example, adding the stochastic term *v*_1_(*t*) to Equation 5 gives

$${\dot{x}}_{1}\left(t\right)=a_{11}x_{1}\left(t\right)+v_{1}\left(t\right).$$ (10)

At each time *t*, the random variable *v*_1_(*t*) provides an endogenous input to the neuronal variable so that *x*_1_(*t*) doesn’t converge to zero despite the negative self-decay rate *a*_11_. This holds true even in the absence of experimental inputs and inputs from other variables, as is the case in Equation 10. In other words, the neuronal activity is now also modeled as an intrinsically fluctuating signal, that is, a stochastic process. The addition of a stochastic term to a dynamical system is traditionally used to model noise, often assumed to be white (that is, serially uncorrelated and with a flat spectral density). Spectral DCM relaxes this assumption and allows the endogenous fluctuations to be temporally correlated, which makes them non-Markovian and smooth. Specifically, their power spectral density is modeled to follow a power-law decay as a function of the frequency *ω*:

$$G_{v_{j}}\left(\omega \right)=\alpha_{v_{j}}\omega^{-\beta_{v_{j}}}.$$ (11)

The parameters *α*_*v*_*j*__ and *β*_*v*_*j*__ determine the amplitude and the decay rate of the power-law and may differ between neuronal regions (*j* = 1, …, *N*). Note that the power-law family includes the flat spectrum (white noise) as a special case where *β*_*v*_*j*__ = 0.

The endogenous fluctuations driving one neuronal variable are assumed to be independent of those driving the others. The result is that the cross-spectral density of the endogenous fluctuations vector **v**(*t*) = [*v*_1_(*t*), …, *v*_*N*_(*t*)]^⊺^ is a diagonal matrix with entries *G*_*v*_*j*__(*ω*) defined according to Equation 11. More precisely, the Fourier transform of **v**(*t*), denoted as the vector ***V***(*ω*), is a multivariate Gaussian random variable with zero mean and diagonal covariance matrix

$$G_{v}\left(\omega \right)=𝔼[V\left(\omega \right)V{\left(\omega \right)}^{†}]=\text{diag}[G_{v_{j}}\left(\omega \right)],\text{for}j=1,\ldots ,N,$$ (12)

where each diagonal entry is the power spectral density of an endogenous fluctuation variable. We will return to this expression when assembling all the elements of the generative model.

### Observation Function

We motivated the use of state-space models by their ability to distinguish between hidden and observed variables. The function that relates the two is known as the *observation function*. Imagine hearing thunder, where the sound (observed variable) is generated by lightning (hidden variable). The role of the observation function is to describe the intensity and delay of the sound based on the distance from the lightning. The specific observation function used in fMRI is the hemodynamic response function (HRF), which links the neuronal activity to the observed BOLD signal. Similarly to the lightning and thunder example, there is a delay between the neuronal activity and the ensuing peak of the BOLD response. The profile of the response depends on several region-specific biophysical parameters and can be modeled mathematically (Stephan, Weiskopf, Drysdale, Robinson, & Friston, 2007). For simplicity, we will denote the HRF of a brain region *j* as *h*_*j*_(*t*), without explicitly indicating the biophysical parameters. The BOLD signal *y*_*j*_(*t*) is obtained via convolution of the HRF with the neuronal activity:

$$y_{j}\left(t\right)=h_{j}\left(t\right)*x_{j}\left(t\right)+e_{j}\left(t\right),$$ (13)

where *j* = 1, …, *N* and *e*_*j*_(*t*) denotes the observation noise. By analogy with the endogenous fluctuations in Equation 11, spectral DCM assumes that the power spectral density of the observation noise also follows a power-law decay:

$$G_{e_{j}}\left(\omega \right)=\alpha_{e_{j}}\omega^{-\beta_{e_{j}}}.$$ (14)

The equivalent representation in vector notation is

$$\begin{array}{l} y\left(t\right)=h\left(t\right)*x\left(t\right)+e\left(t\right),\\ E\left(\omega \right)∼𝒩\left(0,G_{e}\left(\omega \right)\right), \end{array}$$ (15)

where *h*(*t*) is a diagonal matrix with diagonal elements *h*_*j*_(*t*) for *j* = 1, …, *N* (as before, *N* is the number of regions). The noise terms in the vector *e*(*t*) = [*e*_1_(*t*), …, *e*_*N*_(*t*)]^⊺^ are assumed to be independent of each other, that is, the noise in each region is independent of the noise in the other regions. Thus, the Fourier transform of ***e***(*t*), denoted as ***E***(*ω*), is a multivariate Gaussian random variable with zero mean and diagonal covariance matrix 𝔼[***E***(*ω*)***E***(*ω*)^†^] = *G*_*e*_(*ω*), whose diagonal entries are *G*_*e*_*j*__(*ω*), for all *j* = 1, …, *N*. When working in the frequency domain, we can implement the hemodynamic response function as a filter—usually suppressing high frequencies—and implementing delays by operating on the imaginary parts of the Fourier coefficients.

### Putting It All Together

The full state-space model used in spectral DCM is

$$\begin{array}{l} \dot{x}\left(t\right)=Ax\left(t\right)+v\left(t\right),\\ y\left(t\right)=h\left(t\right)*x\left(t\right)+e\left(t\right),\\ V\left(\omega \right)∼𝒩\left(0,G_{v}\left(\omega \right)\right),\\ E\left(\omega \right)∼𝒩\left(0,G_{e}\left(\omega \right)\right), \end{array}$$ (16)

where each equation has been introduced in its respective section above (for simplicity, we have omitted the additional term describing the sampling error of the cross-spectral density; K. J. Friston et al., 2014). Since DCM uses a Bayesian framework, all the model parameters are equipped with a prior distribution (K. J. Friston et al., 2014). Their posterior distribution is then computed via Bayesian inference using variational Laplace (K. J. Friston, Mattout, Trujillo-Barreto, Ashburner, & Penny, 2007; Zeidman et al., 2023). In spectral DCM, this goal is achieved by fitting the generative model in Equation 16 to the cross-spectral density of the data, which is defined as the matrix

$$G_{y}\left(\omega \right)=𝔼[𝓕\left\{y\left(t\right)\right\}𝓕{\left\{y\left(t\right)\right\}}^{†}].$$ (17)

In SPM12, the cross-spectral density is estimated in a parametric fashion, by fitting a vector autoregressive model to the observed time series and leveraging its properties (the default model order used for the slow BOLD fluctuations is 8). Going “backwards” from the observed cross-spectral density to the parameter distribution is only possible once we specify the forward model. Forward generative modelA model that generates the data feature of interest using a set of biologically-plausible parameters. In spectral DCM, the generative model produces the BOLD cross-spectral density using neuronal and hemodynamic parameters. Specifying the forward model means to derive *G*_*y*_(*ω*) as a function of the model parameters. We can start by invoking the convolution theorem, which states that the Fourier transform of a convolution of two functions is the (dot) product of their Fourier transforms. In the case of the observed signal ***y***(*t*), we get

$$\begin{array}{l} 𝓕\left\{y\left(t\right)\right\}=𝓕\left\{h\left(t\right)*x\left(t\right)+e\left(t\right)\right\}\\ \;=𝓕\left\{h\left(t\right)\right\}𝓕\left\{x\left(t\right)\right\}+𝓕\left\{e\left(t\right)\right\}\\ \;=H\left(\omega \right)X\left(\omega \right)+E\left(\omega \right), \end{array}$$ (18)

where the terms in capital letters are the Fourier transforms of the corresponding lowercase functions. Linear control theory gives us a useful expression for ***X***(*ω*), obtained as the solution to the linear differential equation in Equation 1 via the Laplace method:

$$X\left(\omega \right)={\left(i\omega I-A\right)}^{-1}V\left(\omega \right),$$ (19)

where *I* is the *N*-dimensional identity matrix (Pipes, 1968). Plugging Equations 18 and 19 into Equation 17 yields

$$\begin{array}{l} G_{y}\left(\omega \right)=𝔼[H\left(\omega \right)X\left(\omega \right)X{\left(\omega \right)}^{†}H{\left(\omega \right)}^{†}]+𝔼[E\left(\omega \right)E{\left(\omega \right)}^{†}]\\ \;+𝔼[H\left(\omega \right)X\left(\omega \right)]𝔼[E{\left(\omega \right)]}^{†}+𝔼\left[E\left(\omega \right)\right]𝔼{\left[H\left(\omega \right)X\left(\omega \right)\right]}^{†} \end{array}$$ (20)

$$\begin{array}{l} \;=𝔼[H\left(\omega \right){\left(i\omega I-A\right)}^{-}V\left(\omega \right)V{\left(\omega \right)}^{†}{\left(-i\omega I-A^{⊺}\right)}^{-}H{\left(\omega \right)}^{†}]\\ \;+𝔼[E\left(\omega \right)E{\left(\omega \right)}^{†}] \end{array}$$ (21)

$$\;=H\left(\omega \right){\left(i\omega I-A\right)}^{-}G_{v}\left(\omega \right){\left(-i\omega I-A^{⊺}\right)}^{-}H{\left(\omega \right)}^{†}+G_{e}\left(\omega \right).$$ (22)

The last two terms in Equation 20 vanish since the noise term ***E***(*ω*) is a Gaussian random variable with zero mean, that is, 𝔼[***E***(*ω*)] = 0. In Equation 22, we have substituted 𝔼[***V***(*ω*)***V***(*ω*)^†^] = *G*_*v*_(*ω*) and 𝔼[***E***(*ω*)***E***(*ω*)^†^] = *G*_*e*_(*ω*), as defined in the Endogenous Fluctuations and the Observation Function sections.

We have now fully described the forward model used in spectral DCM. The predicted cross-spectral density can be computed using the following model parameters:

(a)  the effective connectivity parameters in the *A* matrix;
(b)  the power-law parameters (i.e., the amplitude and the exponent) describing the spectrum of the endogenous fluctuations and the observation noise (Equations 11 and 14); and
(c)  the observation function parameters, such as the biophysical parameters of the BOLD balloon model.

Crucially, neither the neuronal state variables ***X***(*ω*) nor the endogenous fluctuations ***V***(*ω*) appear in Equation 22, only the parameters describing their cross-spectral densities. This parameterization allows spectral DCM to infer the model parameters listed above without inferring the hidden neuronal states. Inferring the state variables (neuronal time series) is a computationally harder problem addressed by stochastic DCM (Li et al., 2011).

### Simulated and Empirical Cross-Spectral Density

**Figure 3.**
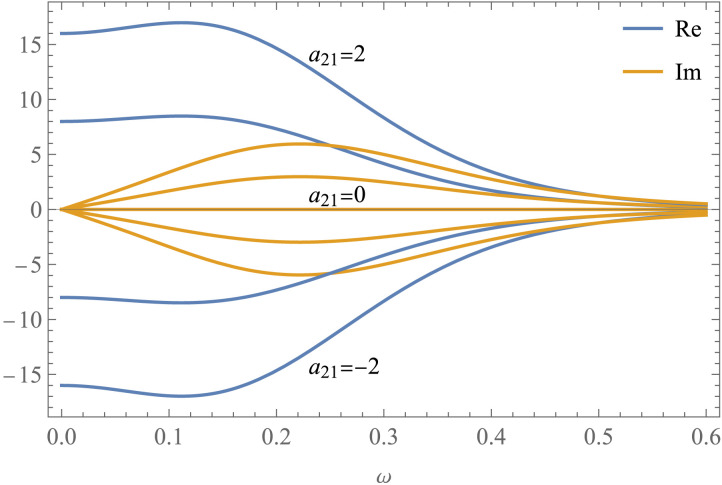
Cross-spectral density of the BOLD signal as a function of the effective connectivity between two neuronal state variables (*a*_21_). In this first example, increasingly large and positive values of *a*_21_ generate increasingly large and positive cross-spectral density amplitudes, while negative values generate negative amplitudes. However, this monotonic relationship doesn’t hold in more realistic scenarios. When *a*_21_ = 0, the two neuronal state variables are independent and the cross-spectral density is zero at all frequencies.

The forward model enables Bayesian inversion and also allows us to understand how different parameters affect the observed cross-spectral density. Figure 3 shows how the cross-spectral density [*G*_*y*_(*ω*)]_21_ varies in a system with two hidden neuronal state variables, as a function of their effective connectivity strength. Specifically, the chosen effective connectivity matrix is

$$A=\left[\begin{array}{cc} -\frac{1}{2} & 0\\ a_{21} & -\frac{1}{2} \end{array}\right].$$ (23)

The asymmetry in *A* indicates a directed effect of the first state variable on the second, but not vice versa. The strength of the connection is determined by *a*_21_. In this first simple example, increasingly large and positive values of *a*_21_ generate increasingly large and positive cross-spectral density amplitudes. Similarly, negative values generate negative amplitudes (but we’ll soon encounter more complex scenarios that violate this monotonic relationship). When *a*_21_ = 0, the two neuronal state variables are independent of each other and the cross-spectral density is zero at all frequencies (Figure 3; also see the Supporting Information for an explanation of the real and imaginary parts). Here, the cross-spectral densities of the endogenous fluctuations and of the observation noise are identical to each other and identical for both state variables (so that both diagonal entries are equal):

$$G_{v}\left(\omega \right)=G_{e}\left(\omega \right)=\left[\begin{array}{cc} \frac{1}{{\left(1+\omega \right)}^{2}} & 0\\ 0 & \frac{1}{{\left(1+\omega \right)}^{2}} \end{array}\right]={\left(1+\omega \right)}^{-2}I.$$ (24)

The observation function is also identical for both state variables. Spectral DCM employs an HRF model with region-specific biophysical parameters (Stephan et al., 2007). However, for simplicity, the examples in this section are based on the canonical HRF gamma-mixture, whose Fourier spectrum is

$$H\left(\omega \right)=\frac{6{\left(i\omega +1\right)}^{10}-1}{5{\left(i\omega +1\right)}^{16}}\;I.$$ (25)

High frequencies are “cut off” since the slow HRF smooths the faster neuronal activity (a typical feature of the BOLD signal).

**Figure 4.**
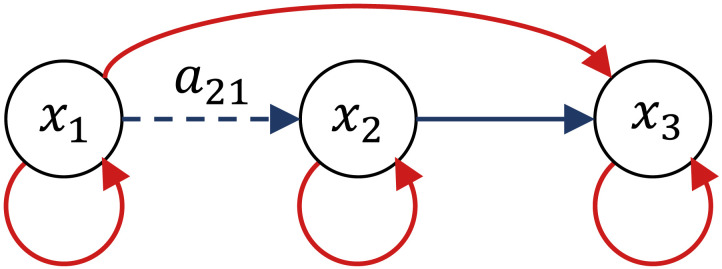
Network graph of the system described by the effective connectivity matrix *A* in Equation 26. The strength of the directed connection from *x*_1_ to *x*_2_ is set via the parameter *a*_21_. The third neuronal state variable receives an inhibitory influence from *x*_1_ and an excitatory influence from *x*_2_. All three variables have the same negative self-connections represented by the diagonal elements of *A*.

Let’s now extend the system in Equation 23 by adding a third neuronal state variable (see Figure 4) and a third row and column to the effective connectivity matrix:

$$A=\left[\begin{array}{ccc} -\frac{1}{2} & 0 & 0\\ a_{21} & -\frac{1}{2} & 0\\ -\frac{1}{2} & \frac{3}{2} & -\frac{1}{2} \end{array}\right].$$ (26)

**Figure 5.**
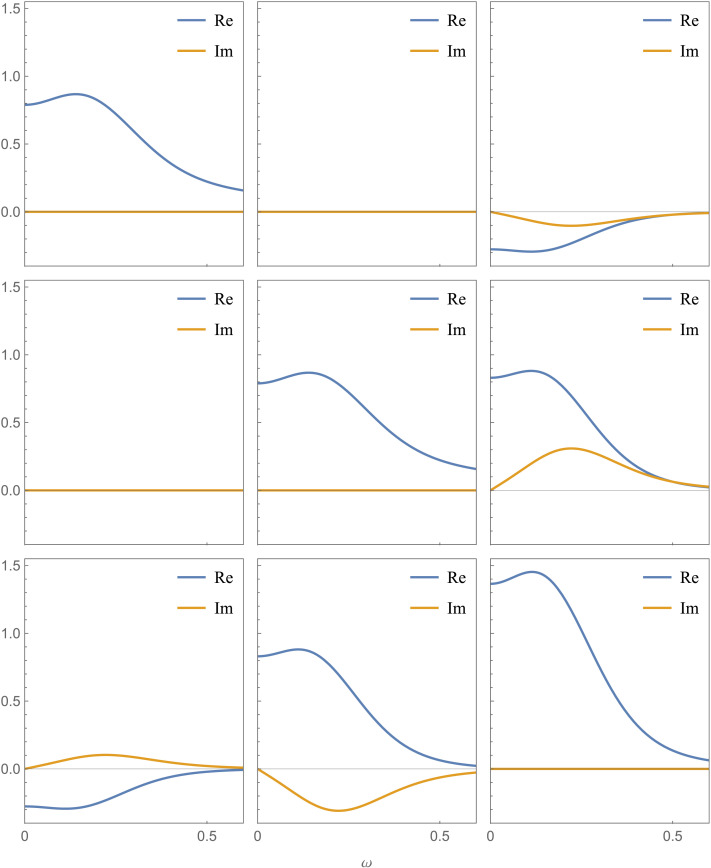
Simulated BOLD cross-spectral density of the system in Figure 4, generated via the forward model in Equation 22. The effective connectivity matrix is presented in Equation 26. This figure shows only the special case where *a*_21_ = 0, but using different values of *a*_21_ would generate different sets of nine plots. Each plot corresponds to a pair of state variables and, if we chose a specific value for the frequency *ω*, we would obtain a 3 × 3 matrix (the cross-spectral density matrix at that specific frequency). The three plots on the diagonal represent the power spectral densities, which take real values (zero imaginary part). The same canonical hemodynamic response function defined in Equation 25 is used for all variables in this example, although spectral DCM employs the BOLD balloon model with region-specific biophysical parameters. The power-law parameter of the endogenous fluctuations and of the observation noise are set as in Equation 24. For simplicity, we have normalized the rows and columns of the cross-spectral density so that integrating over all frequencies directly generates the correlation matrix instead of the covariance matrix.

As before, the strength of the directed connection from *x*_1_ to *x*_2_ is set via the parameter *a*_21_. The third neuronal state variable receives an inhibitory influence from *x*_1_ and an excitatory influence from *x*_2_. All three state variables have the same negative self-connections represented by the diagonal elements of *A*. The resulting cross-spectral density is plotted in Figure 5, assuming the same canonical HRF defined in Equation 25 and the same power-law parameters as in Equation 24. The figure shows only the special case where *a*_21_ = 0, but different values of *a*_21_ would generate different sets of nine plots.

**Figure 6.**
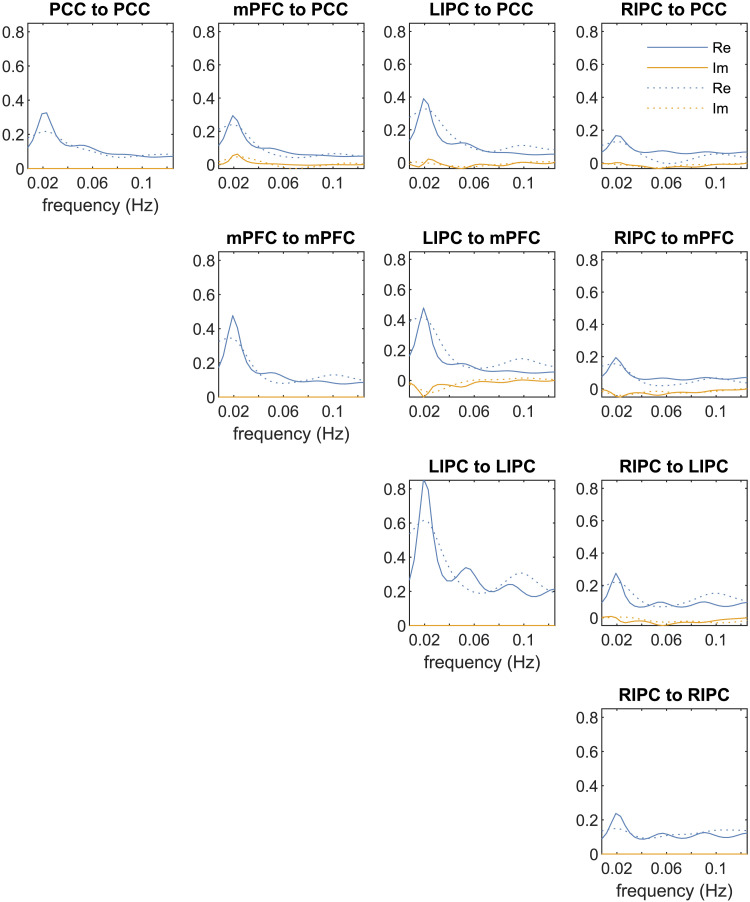
BOLD cross-spectral density obtained via spectral DCM analysis of a real resting-state fMRI dataset. The dashed lines represent the predicted cross-spectral density and the solid lines the observed ones. Reproduced with permission from Chapter 38 of the SPM12 manual (Ashburner et al., 2020).

It is instructive to qualitatively compare the simulated cross-spectral density plots in Figure 5 with the empirical plots in Figure 6, obtained by fitting the spectral DCM model to a real resting-state dataset (Razi et al., 2015). Note that the empirical plots correspond to a system with four neuronal variables instead of three. Here, a four-region default mode network is modeled with posterior cingulate cortex (PCC), medial prefrontal cortex (mPFC), and bilateral inferior parietal cortices (IPC) as its nodes. We used a fully connected model where each state variable (or node) is connected to every other state variable. The figure is reproduced from Chapter 38 of the SPM12 manual (Ashburner et al., 2020), which provides the link to the data and a step-by-step tutorial to replicate the DCM specification and estimation results using the SPM graphical interface. Despite the clear differences due to different data and parameter settings, the empirical cross-spectral density plots also feature a single large peak at low frequencies, followed by a decay at larger frequencies (with smaller fluctuations). If the data are too noisy, owing to head motion and various physiological artifacts, additional large peaks may appear at higher frequencies. These empirical plots can be visualized using the review function (spm_dcm_fmri_csd_results()) in SPM. Another DCM diagnostics function (spm_dcm_fmri_check()) in SPM also reports the percentage of variance explained (*R*^2^), which is a useful performance metric to judge the quality and success of the model fit to the data (see Figure S2 in the Supporting Information).

## EFFECTIVE AND FUNCTIONAL CONNECTIVITY

The main difference between effective and functional connectivity is that the former characterizes the interaction between neuronal state variables, while the latter describes statistical dependence between the observed variables (e.g., the BOLD signals in fMRI). Given its logical and biological precedence, effective connectivity can be used to derive functional connectivity. Specifically, the functional connectivity matrix of the observed time series can be obtained by integrating the cross-spectral density over all frequencies. The reason will become clear in the following sections, and a mathematical proof will be given in Equation 37. Let’s first return to the simulated example depicted in Figure 4 to understand the relevant implications. The effective connectivity matrix of the system is given in Equation 26. Integrating the cross-spectral density in Figure 5 over all frequencies gives us the 3 × 3 symmetric correlation matrix *R*, typically used to quantify the functional connectivity:

$$R=\left[\begin{array}{ccc} 1 & \rho_{21} & \rho_{31}\\ \rho_{21} & 1 & \rho_{32}\\ \rho_{31} & \rho_{32} & 1 \end{array}\right].$$ (27)

**Figure 7.**
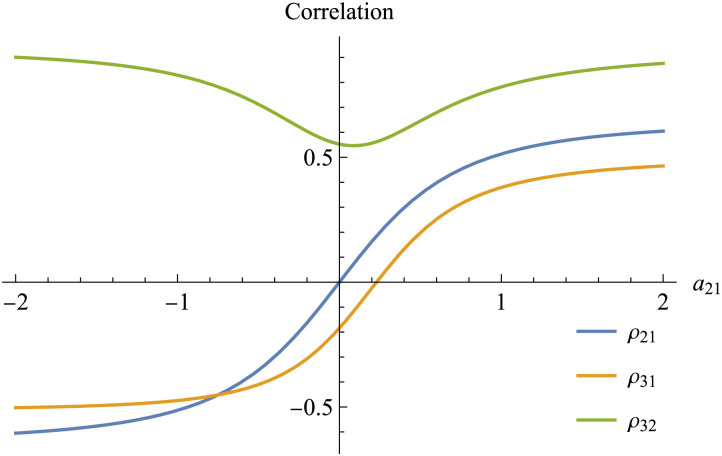
Simulated BOLD functional connectivity of the system in Figure 4, as a function of the effective connectivity parameter *a*_21_. The three curves corresponding to the correlation values are obtained analytically by integrating the cross-spectral density shown in Figure 5 over all frequencies. Changing a single effective connectivity parameter, *a*_21_, has a global impact across all pairwise functional connectivity values in the network. Both correlations *ρ*_21_ and *ρ*_31_ increase monotonically with *a*_21_, but this is not the case for *ρ*_32_. Therefore, there is no one-to-one mapping between effective and functional connectivity that holds in general.

The nine integrals produce real numbers because all the imaginary parts are “odd functions” so their integrals are equal to zero (see the Supporting Information for a discussion of even and odd functions and their Fourier transforms). Crucially, all three pairwise correlations (*ρ*_21_, *ρ*_31_, *ρ*_32_) explicitly depend on the effective connectivity parameter *a*_21_, as illustrated in Figure 7 (for the analytic solutions, see Equation S3 in the Supporting Information).

This shows that a local variation in a single effective connectivity parameter in the *A* matrix can have a global impact across all functional connectivity values in the network. Let’s examine each pair to unpack some of the many nuances involved. First, the symmetric nature of the correlation matrix requires that *ρ*_*kj*_ = *ρ*_*jk*_, even if *a*_*kj*_ ≠ *a*_*jk*_. This is a key difference between functional and effective connectivity: Only the latter is directed and is able to differentiate between two bilateral connections.

Both correlations *ρ*_21_ and *ρ*_31_ increase monotonically with *a*_21_, but this is not the case for *ρ*_32_. Therefore, there is no one-to-one mapping between effective and functional connectivity that holds in general (Park & Friston, 2013). This poses a challenge for spectral DCM because it makes model fitting an ill-posed problem with multiple potential solutions. Technically, this problem is mitigated by using the cross-spectral density that implicitly contains information about functional connectivity over all lags (we will unpack this below) and by using priors on the solutions implicit in the functional form of the DCM. Although model fitting remains an ill-posed problem, these two additional constraints allow spectral DCM to find better solutions, such that the model can reproduce a larger set of statistical relationships between the observed time series. One way to appreciate the amount of additional information and constraints provided by the cross-spectral density over the zero-lag correlation is to inspect Figure 5 again. Zero-lag correlation measures only the area under the curves in the nine plots, regardless of their detailed shapes. On the other hand, spectral DCM fits all values taken by the cross-spectral density curves at different frequencies. This requirement narrows the parameter space in a useful way (note that the area under the curve is still reproduced as a consequence).

The sign of *ρ*_31_ (representing a positive or a negative correlation) either matches or contradicts the sign of the underlying effective connectivity *a*_31_ (representing an excitatory or an inhibitory connectivity), depending on how we set *a*_21_. There is a value of *a*_21_ such that *ρ*_31_ = 0, even though the underlying effective connectivity *a*_31_ is negative (i.e., inhibitory). Again, this shows that each pairwise functional connectivity value is a summary statistic (global property) of the system: Given a pair of variables *j* and *k*, the correlation value *ρ*_*kj*_ depends not only on the corresponding effective connectivity parameter *a*_*kj*_ but also, potentially, on all the entries of the *A* matrix.

While here we have discussed its dependence on only effective connectivity, functional connectivity also depends on the parameters characterizing the endogenous fluctuations, the observation function, and the observation noise. This is because all these parameters appear in the forward model for the cross-spectral density derived in Equation 22 and, in turn, determine the correlation matrix of the system. In fMRI, specific and reproducible spatial patterns of functional connectivity could simply arise from specific and reproducible variations in the hemodynamic response across brain regions—even in the absence of interregional effective connectivity (Rangaprakash, Wu, Marinazzo, Hu, & Deshpande, 2018). When comparing groups, differences in BOLD functional connectivity may reflect differences in the vasculature rather than in effective connectivity (or both), for example because of aging or a neurodegenerative or psychiatric disease (Tsvetanov, Henson, & Rowe, 2020). This important point is further discussed in K. J. Friston (2011), where it is shown that the correlation values are also influenced by different levels of observation noise:

One can see a change in correlation by simply changing the signal-to-noise ratio of the data. This can be particularly important when comparing correlations between different groups of subjects. For example, obsessive compulsive patients may have a heart rate variability that differs from normal subjects. This may change the noise in observed hemodynamic responses, even in the absence of neuronal differences or changes in effective connectivity. (p. 22)

In fact, separating these confounding factors from the effective connectivity was one of the main motivations for the development of DCM. This is not to say that DCM is without issues: Fitting a large model with many parameters—using the limited amount of data available in typical fMRI studies—is not guaranteed to produce optimal estimates. We’ll discuss this and other limitations in the Assumptions and Limitations section.

Functional connectivity faces yet another challenge. Even though we observed that any variations in effective connectivity can propagate through the network and affect the correlation among several brain areas, one could hope that the pair of variables showing the largest functional connectivity change would coincide with the pair evincing the largest effective connectivity change. Alas, this is also not guaranteed. When *a*_21_ increases from zero to one, *R* shows the following changes:

$$\Delta R=\left[\begin{array}{ccc} 0 & 0.513367 & 0.379316\\ 0.513367 & 0 & 0.782089\\ 0.379316 & 0.782089 & 0 \end{array}\right].$$ (28)

The largest functional connectivity change is observed in *ρ*_32_, not in *ρ*_21_ as we might have expected. The fact that the variable pairs showing the largest changes in functional connectivity do not necessarily coincide with the pairs with the largest changes in effective connectivity may hinder the use of functional connectivity as a quick and easy way for selecting regions of interest in DCM studies.

On the other hand, functional connectivity has proven valuable for fingerprinting, that is, identifying an individual based on their brain activity (Finn et al., 2015). It is also useful in studies aiming to differentiate between two groups or conditions (by detecting statistically significant changes in correlation between observed time series), rather than in identifying which effective connections between brain regions underlie that change. Moreover, being a global property of the system, each pairwise functional connectivity value naturally captures higher order interactions, which is a topic of growing interest in complex systems, network science, and neuroimaging (Benson, Gleich, & Leskovec, 2016; Rosas et al., 2022).

So far, we have examined only the zero-lag correlation matrix because it is widely used to quantify the functional connectivity in neuroimaging. However, spectral DCM doesn’t explain just the zero-lag correlation but also the cross-correlations between all variables at all time lags. To understand this point, in the next section, we will invoke the elegant Wiener-Khinchin theorem, which links the cross-covariance function to the cross-spectral density.

### Autocovariance and Cross-Covariance Functions

Let’s briefly revisit the role of the self-connections in the presence of the endogenous fluctuations. As discussed in the Effective Connectivity section, a self-connection determines the rate of decay of a variable. It can also be understood as quantifying the memory of a variable, that is, whether inputs have a short-lived or long-lasting impact on its activity. Large negative self-connections reflect short memory: The variable “forgets” its past quickly and responds promptly to any new inputs. On the contrary, small negative values reflect long memory: The neuronal variable integrates and smooths out the endogenous fluctuations and other inputs, resulting in slower oscillations and lower frequencies.

The concept of a system having memory can be quantified via the *autocovariance function*. For a deterministic signal *z*(*t*), there is a simple intuition: The autocovariance function at a time lag Δ*t* measures the similarity (i.e., sample covariance) between the time series *z*(*t*) and a shifted version of itself, that is, *z*(*t* + Δ*t*). The agreement is perfect when there is no shift (Δ*t* = 0) and it typically decreases with longer time lags, unless the signal is constant or periodic. However, spectral DCM is concerned with stochastic (nondeterministic) processes, as we discussed in the Power Spectral Density and Cross-Spectral Density section and illustrated in Figure S1 in the Supporting Information. Let’s consider the single stochastic neuronal variable *x*_1_. At any given time point, *x*_1_(*t*) is not a number but a random variable. If, after a time interval Δ*t*, the random variable *x*_1_(*t* + Δ*t*) is still positively correlated with its previous state *x*_1_(*t*), the autocovariance between the two time points would be positive. If, as the time interval further increases, *x*_1_ forgets its past state and becomes independent of it, the autocovariance would become zero at that point (for BOLD signals, time intervals or time lags are always multiples of the repetition time). In summary, the autocovariance function measures the covariance between the states of the same stochastic process at two different points in time. For the stationary processes considered here, the autocovariance only depends on the time interval Δ*t*:

$$\sigma_{11}\left(\Delta t\right)=\mathrm{cov}\left[x_{1}\left(t\right),x_{1}\left(t+\Delta t\right)\right].$$ (29)

Dividing the autocovariance function by *σ*_11_(0) would produce the commonly used *autocorrelation function*, whose values are normalized to the [−1, 1] interval.

We can compute the autocovariance explicitly in the simple scenario of Equation 10, where *x*_1_ is driven by endogenous fluctuations only, that is, it doesn’t receive any other inputs. For simplicity, assume that the endogenous fluctuations were modeled as a white noise process with *α*_*v*_1__ = 1 and *β*_*v*_1__ = 0. The equation would then describe an Ornstein-Uhlenbeck process that has the following autocovariance function (Vatiwutipong & Phewchean, 2019):

$$\sigma_{11}\left(\Delta t\right)=\frac{e^{a_{11}|\Delta t|}}{-2a_{11}}.$$ (30)

This confirms the intuition above: Large negative values of *a*_11_ correspond to short memory; in this case, the decay is exponential.

There is another reason why the autocovariance function is relevant for spectral DCM and, more generally, for DCM applications to electroencephalography (EEG) and magnetoencephalography (MEG). These models and modalities are concerned with the power spectral density of signals, and the Wiener-Khinchin theorem proves that the power spectral density of a stationary process is the Fourier transform of its autocovariance function. In the case of *x*_1_, we get

$$G_{x_{1}}\left(\omega \right)=𝓕\left\{\sigma_{11}\left(\Delta t\right)\right\}=𝓕\left\{\frac{e^{a_{11}|\Delta t|}}{-2a_{11}}\right\}=\frac{1}{a_{11}^{2}+\omega^{2}}.$$ (31)

**Figure 8.**
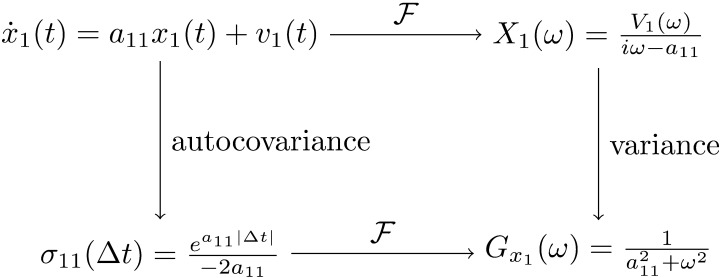
The two equivalent representations of the power spectral density. Starting from the Ornstein-Uhlenbeck process as an example (top left), the power spectral density can be computed either as the variance of the Fourier transform (top right, then bottom right), or as the Fourier transform of the autocovariance function (bottom left, then bottom right). The former uses the definition of the power spectral density; the latter uses the Wiener-Khinchin theorem. In the Ornstein-Uhlenbeck process, the endogenous fluctuations (*v*_1_(*t*)) are modeled as a white noise process. Its Fourier transform, denoted as *V*_1_(*ω*), is a normal random variable for each frequency *ω*.

This offers an alternative way to compute the power spectral density compared with the definition given in the Power Spectral Density and Cross-Spectral Density section. The two equivalent representations are often portrayed with arrow diagrams in spectral DCM papers (K. J. Friston et al., 2014; Razi & Friston, 2016). Here, a similar diagram is provided in Figure 8 using the Ornstein-Uhlenbeck process as an example.

In the presence of multiple state variables, the autocovariance function is replaced by the more general *cross-covariance function*

$$\Sigma_{x}\left(\Delta t\right)=\mathrm{cov}\left(x\left(t\right),x\left(t+\Delta t\right)\right).$$ (32)

For any time lag Δ*t*, the cross-covariance function produces an *N* × *N* matrix with entries

$${\left[\Sigma_{x}\left(\Delta t\right)\right]}_{jk}=\sigma_{jk}\left(\Delta t\right)=\mathrm{cov}\left(x_{j}\left(t\right),x_{k}\left(t+\Delta t\right)\right).$$ (33)

Each diagonal element in this matrix is the autocovariance function of a single neuronal variable, introduced in Equation 29. The off-diagonal elements are the cross-covariance functions between different state variables. For deterministic processes, the visual intuition is analogous to the autocovariance case: For each pair of variables *x*_*j*_ and *x*_*k*_, the cross-covariance function *σ*_*jk*_(Δ*t*) measures the similarity between the time series *x*_*j*_(*t*) and a shifted version of *x*_*k*_ by a time lag Δ*t*. The analogy with memory can then be used to extend the intuition to stochastic processes. When there is no time lag (Δ*t* = 0), the cross-covariance function produces the covariance matrix Σ_*x*_>(0), which is a symmetric matrix because *σ*_*jk*_(0) = *σ*_*kj*_(0). After normalization, this yields the correlation matrix that is used to quantify the functional connectivity in neuroimaging.

### Cross-Spectral Density and Functional Connectivity

We can again invoke the Wiener-Khinchin theorem and obtain the cross-spectral density as the Fourier transform of the cross-covariance function:

$$G_{x}\left(\omega \right)=𝓕\left\{\Sigma_{x}\left(\Delta t\right)\right\}.$$ (34)

We previously noted that the off-diagonal elements of the cross-spectral density matrix are complex numbers and that their definition provided in Equation 9 doesn’t lend itself to an intuitive understanding. However—now armed with the Wiener-Khinchin theorem—we gain another perspective. Each off-diagonal element is equivalent to the Fourier transform of the cross-covariance function between two variables. By modeling the cross-spectral density, spectral DCM doesn’t capture just the zero-lag correlations but also the cross-correlations between all variables at all time lags. This is true for both the hidden neuronal variables and the observed (e.g., BOLD) variables. The equivalent of Equation 34 for the observed variables is

$$G_{y}\left(\omega \right)=𝓕\left\{\Sigma_{y}\left(\Delta t\right)\right\}.$$ (35)

We opened the Effective and Functional Connectivity section by stating that the functional connectivity matrix can be obtained by integrating the cross-spectral density over all frequencies. To understand why, we need to invert Equation 35 using the (inverse) Fourier transform,

$$\Sigma_{y}\left(t\right)={𝓕}^{-1}\left\{G_{y}\left(\omega \right)\right\}=\int_{-\infty }^{\infty }G_{y}\left(\omega \right)e^{i\omega t}\text{d}\omega ,$$ (36)

which retrieves the cross-covariance function from the cross-spectral density *G*_*x*_(*ω*). When *t* = 0, we have the desired result:

$$\Sigma_{y}\left(0\right)={𝓕}^{-1}\left\{G_{y}\left(\omega \right)\right\}=\int_{-\infty }^{\infty }G_{y}\left(\omega \right)\text{d}\omega .$$ (37)

The correlation matrix *R* is finally obtained by normalizing the covariance matrix. This concludes our treatment of the cross-spectral density. We hope that the second perspective provided in this section will help the reader form a fuller picture of this central concept for spectral DCM.

## ASSUMPTIONS AND LIMITATIONS

### State-Space Modeling

The foundational hypothesis of the DCM framework is that the system of interest can be modeled using a state-space approach. This formulation separates the equations describing the temporal evolution of unobserved variables (e.g., the neuronal activity) from those describing the observed variables (e.g., the BOLD). Such a separation is important because some of the model parameters have a direct interpretation in terms of effective connectivity between unobserved neuronal populations rather than statistical dependency between observed variables. A subtle and important consequence of the distinction between functional effective connectivity is that one can only estimate recurrent or self-inhibition using a state-space model. This is because the correlation of a time series with itself is always 1 (and the variance is always positive). Assessing self-connectivity in terms of excitability or disinhibition of a node can be empirically important (e.g., in estimating changes in excitation-inhibition balance or condition-specific changes in the “gain” of a particular source or region).

### Continuous-Time Formulation

Like most DCM approaches, spectral DCM treats time as a continuous quantity. Representing time as a sequence of discrete steps may seem more natural, especially for fMRI, where the observations are recorded at evenly spaced time intervals with a relatively low sampling rate. However, DCM also models the neuronal activity, which unfolds at a much faster timescale and in an asynchronous manner. These two features can be naturally modeled using differential equations in continuous time. The discrete formulation usually converges to the continuous one with faster acquisition times, as is the case for EEG and MEG.

### Separation of Timescales and Macroscopic Modeling

Spectral DCM assumes that a single macroscopic neuronal variable can capture the essential dynamical properties of a population of neurons. This is not just a pragmatic way to avoid billions of equations representing individual neurons. It is an approach based on the separation of timescales that has a long history in dynamical systems theory (Carr, 1981; Haken, 1983). The assumption is that the neuronal activity can be separated into slow and fast modes: The fast modes decay quickly so that the long-term behavior of the system can be described by a few slow modes, or even a single one. Mathematically, the assumption (often satisfied in real systems) is that only a small number of eigenvalues of the Jacobian are near zero, while the rest are large and negative (K. J. Friston et al., 2021; K. J. Friston, Li, Daunizeaue, & Stephan, 2011). Macroscopic modeling also relies on the mean-field assumption that the dynamics of one region are determined by the mean activity in another (Deco, Jirsa, Robinson, Breakspear, & Friston, 2008). Since the mean activity is dominated by the slow modes, it is possible to build a compact macroscopic model where only the slow modes are communicated among brain regions, whereas the fast endogenous fluctuations affect only the local activity. This is precisely the neuronal model in Equation 1. It is important to note that, despite being grounded in dynamical and complex systems theory, this model is an abstraction. Biological details are necessarily omitted to enable the analytic treatment and faster numerical computations. A detailed critical review of the biophysical and statistical foundations of DCM is provided in Daunizeau, David, and Stephan (2011).

### Stationarity

Among the DCM variants, spectral DCM has the most direct conceptual and analytical links to functional connectivity, which we have examined in the Effective and Functional Connectivity section. Both methods can be read as assuming that the observed processes are weakly stationary, that is, that their covariance remains unchanged over the length of the experiment (Liégeois, Laumann, Snyder, Zhou, & Yeo, 2017). In the case of functional connectivity, this assumption arises because the covariance is used as a proxy for connectivity, after being normalized to obtain the correlation matrix. Therefore, assuming weak stationarity is equivalent to assuming that the functional connectivity remains unchanged. Similarly, in spectral DCM, the stationarity assumption allows one to interpret the effective connectivity as remaining unchanged over the length of the time series (K. J. Friston et al., 2014; Moran et al., 2009). This is because the effective connectivity is inferred from the observed cross-spectral density, that is, the Fourier transform of the cross-covariance function (see the Cross-Spectral Density and Functional Connectivity section), which remains unchanged under stationarity assumptions. However, in contrast to functional connectivity, spectral DCM only treats the cross-covariance of the observed time series as a means to an end, where the end is to infer the effective connectivity between neuronal variables (and various other model parameters). In other words, spectral DCM looks “under the hood” of functional connectivity and beyond the observed variables.

The stationarity assumption can certainly be challenged, and it is generally untenable in task experiments; nonetheless, it is widely adopted in resting-state studies. Stationarity is typically assumed not just in functional connectivity and spectral DCM, but also in Granger causality analysis (Granger, 1969; Seth, Chorley, & Barnett, 2013), transfer entropy (Bossomaier, Barnett, Harré, & Lizier, 2016), and autoregressive modeling (Liégeois et al., 2017). That said, Granger causality and information-theoretic methods have been adapted for nonstationary processes (Dhamala, Rangarajan, & Ding, 2008; Gómez-Herrero et al., 2015; Lizier, Prokopenko, & Zomaya, 2008; Paluš, 2018; Wollstadt, Martínez-Zarzuela, Vicente, Díaz-Pernas, & Wibral, 2014), and time-varying approaches to functional connectivity analysis have been rapidly gaining popularity (Lurie et al., 2020; Novelli & Razi, 2022). A time-varying extension of spectral DCM has also been developed (Park, Friston, Pae, Park, & Razi, 2018). All of these methods relax the stationarity assumption and allow the statistical properties of the system to change over the course of the experiment. Practically, in DCM, one appeals to something called an adiabatic approximation: that effective connectivity is constant over a small timescale but can change at longer timescales. This means that one can apply spectral DCM to short segments of data and then examine (or model) slow fluctuations in effective connectivity (Jafarian, Zeidman, Wykes, Walker, & Friston, 2021; Rosch et al., 2019; Zarghami & Friston, 2020).

### Linearity

The second assumption that spectral DCM (partially) shares with functional connectivity is linearity, although nonlinear extensions of both methods exist (Kraskov, Stögbauer, & Grassberger, 2004; Stephan et al., 2008). In DCM for fMRI, the use of a linear random differential equation to model the neuronal activity is motivated by the separation of timescales, whereby the neuronal variables represent the slow modes of the system, which are assumed to be linearly coupled, while the endogenous random fluctuations represent the fast modes (K. J. Friston et al., 2011). Spectral DCM also extends the linearity assumption to the observation function, which can be a nonlinear function of time and frequency (as in Equation 25) but is linearly convolved with the neuronal activity (Equation 13). In the Endogenous Fluctuations section, we have observed that deterministic linear models have a limited scope since they can only describe a system that converges to equilibrium or generates a sequence of identical oscillations. Adding stochastic terms to the linear differential equations allows for a richer repertoire of behaviors.

### Stochastic Fluctuations

The addition of a stochastic term to a dynamical system is traditionally used to model noise, often assumed to be white and temporally uncorrelated. The underlying assumption is that the noise is due to physical processes operating at a much faster timescale than the state variables, such as microscopic thermal fluctuations. Spectral DCM relaxes this assumption and allows the endogenous fluctuations to be temporally correlated, with a spectrum following a power-law decay in the frequency domain (in the Endogenous Fluctuations section, we saw how this form includes white noise as a special case). This is easily motivated by noting that the endogenous fluctuations are themselves generated by dynamical processes within the source or region of interest. The ensuing temporal autocorrelation makes the endogenous fluctuations differentiable and smooth, in line with their characterization as mixtures of fast modes of a dynamical system (K. J. Friston et al., 2011).

### Gaussianity

In line with most DCM approaches, spectral DCM assumes a Gaussian distribution for the prior over model parameters (the nonnegative parameters are transformed using the natural logarithm and are assumed to follow a log-normal distribution). This enables a fast Bayesian inversion scheme called variational Laplace (K. Friston et al., 2007; Zeidman et al., 2023). As for most of the assumptions listed in this section, the Gaussian hypothesis can also be relaxed and other (albeit more computationally intensive) inversion schemes can be used instead, such as Markov chain Monte Carlo methods (Aponte et al., 2022; K. Friston et al., 2007; Xie, Zhang, & Zhao, 2023).

### Many-to-One Mapping

There is no one-to-one mapping between effective and functional connectivity (or cross-spectral density) that holds in general. This is a challenge for spectral DCM because it makes model inversion an ill-posed problem with multiple potential solutions, in the absence of any constraints on the way data are generated. As with all ill-posed problems, this issue is addressed by placing prior constraints on the explanations in the form of a model and prior densities over model parameters. When one does not know which priors to use, a weighted average of plausible priors is often performed in DCM analysis using Bayesian model averaging (Hoeting, Madigan, Raftery, & Volinsky, 1999), where each set of priors corresponds to a separate model. A related challenge is that one cannot always use functional connectivity to identify the regions of interest to study using DCM. As we saw in the Effective and Functional Connectivity section, not even the regions showing the largest differences in correlation are guaranteed to coincide with the regions with a change in effective connectivity.

### Computational Complexity

Despite the simplifying assumptions mentioned above, the computational complexity of spectral DCM limits the number of brain regions that can be studied in reasonable time. Selecting the regions of interest requires more upfront work than in functional connectivity analysis, which can be quickly performed across the whole brain. Given the large size of the parameter space, the specification of the model has been noted as a conceptual issue for DCM in the past (K. J. Friston, Moran, & Seth, 2013). That said, a more recent theoretical advance now enables the exploration of a large model space using Bayesian model reduction (Seghier & Friston, 2013). Alternatively, one can introduce further assumptions to winnow the parameter space. Using functional connectivity to place prior constraints on the eigenvectors of the effective connectivity matrix enables spectral DCM analyses with dozens of brain regions (Razi et al., 2017). In fMRI, ignoring the spatial variability of the hemodynamics and removing the separation between hidden and observed variables leads to the *regression DCM* scheme, which can analyze hundreds of regions in minutes (Frässle et al., 2021). However, this method forgoes the state-space formulation and can be understood as a Bayesian multivariate regression in the frequency domain.

## ACKNOWLEDGMENTS

Adeel Razi is affiliated with the Wellcome Centre for Human Neuroimaging supported by core funding from Wellcome (203147/Z/16/Z). Adeel Razi is a CIFAR Azrieli Global Scholar in the Brain, Mind & Consciousness Program. Karl Friston is funded by the Wellcome Centre for Human Neuroimaging (Ref: 205103/Z/16/Z) and a Canada-UK Artificial Intelligence Initiative (Ref: ES/T01279X/1).

## SUPPORTING INFORMATION

Supporting information for this article is available at https://doi.org/10.1162/netn_a_00348.

## AUTHOR CONTRIBUTIONS

Leonardo Novelli: Conceptualization; Formal analysis; Investigation; Methodology; Software; Visualization; Writing – original draft; Writing – review & editing. Karl Friston: Conceptualization; Methodology; Writing – review & editing. Adeel Razi: Conceptualization; Funding acquisition; Methodology; Supervision; Writing – review & editing.

## FUNDING INFORMATION

Leonardo Novelli, Australian Research Council, Award ID: DP200100757. Adeel Razi, Australian Research Council, Award ID: DE170100128. Adeel Razi, Australian Research Council (https://dx.doi.org/10.13039/501100000923), Award ID: DP200100757. Adeel Razi, Australian National Health and Medical Research, Award ID: 1194910. Karl Friston, Wellcome Centre for Human Neuroimaging, Award ID: 205103/Z/16/Z. Karl Friston, Canada-UK Artificial Intelligence Initiative, Award ID: ES/T01279X/1.

## Supplementary Material



## TECHNICAL TERMS

SPM: The *Statistical Parametric Mapping* open software is widely used for dynamic causal modeling.
Endogenous fluctuations: Intrinsic stochastic fluctuations that serve as a proxy for thoughts or mind-wandering-like processes during resting-state brain activity.
Effective connectivity: The directed effect of one brain region on another, measured as a rate of change.
Forward generative model: A model that generates the data feature of interest using a set of biologically plausible parameters. In spectral DCM, the generative model produces the BOLD cross-spectral density using neuronal and hemodynamic parameters.
Stochastic process: A sequence of random variables, typically indexed by time.
