# Supplementary material for: Spectral dynamic causal modeling: A didactic introduction and its relationship with functional connectivity
Source: Netw Neurosci. 2024 Apr 1;8(1):178–202. doi: 10.1162/netn_a_00348 (PMC10898785; doi:10.1162/netn_a_00348)
Supplement: Supplementary file 1 [file netn-8-1-178-s001.pdf]

SUPPLEMENTARY MATERIALS

STOCHASTIC PROCESSES

A stochastic process is a sequence of random variables. If x is a stochastic process indexed by time, then $x(t)$ is not a single number but a random variable with a given probability distribution. Intuitively, the process is stationary if, when we collect many realisations (known as a statistical *ensemble*) and plot a histogram of their values at different time points, we will obtain the same distribution. Fig. S1 illustrates this concept using a stationary Ornstein-Uhlenbeck process and showing two histograms of the ensemble at two different time points. Although the individual realisations (curves) take different values, they collectively preserve the same Gaussian distribution.

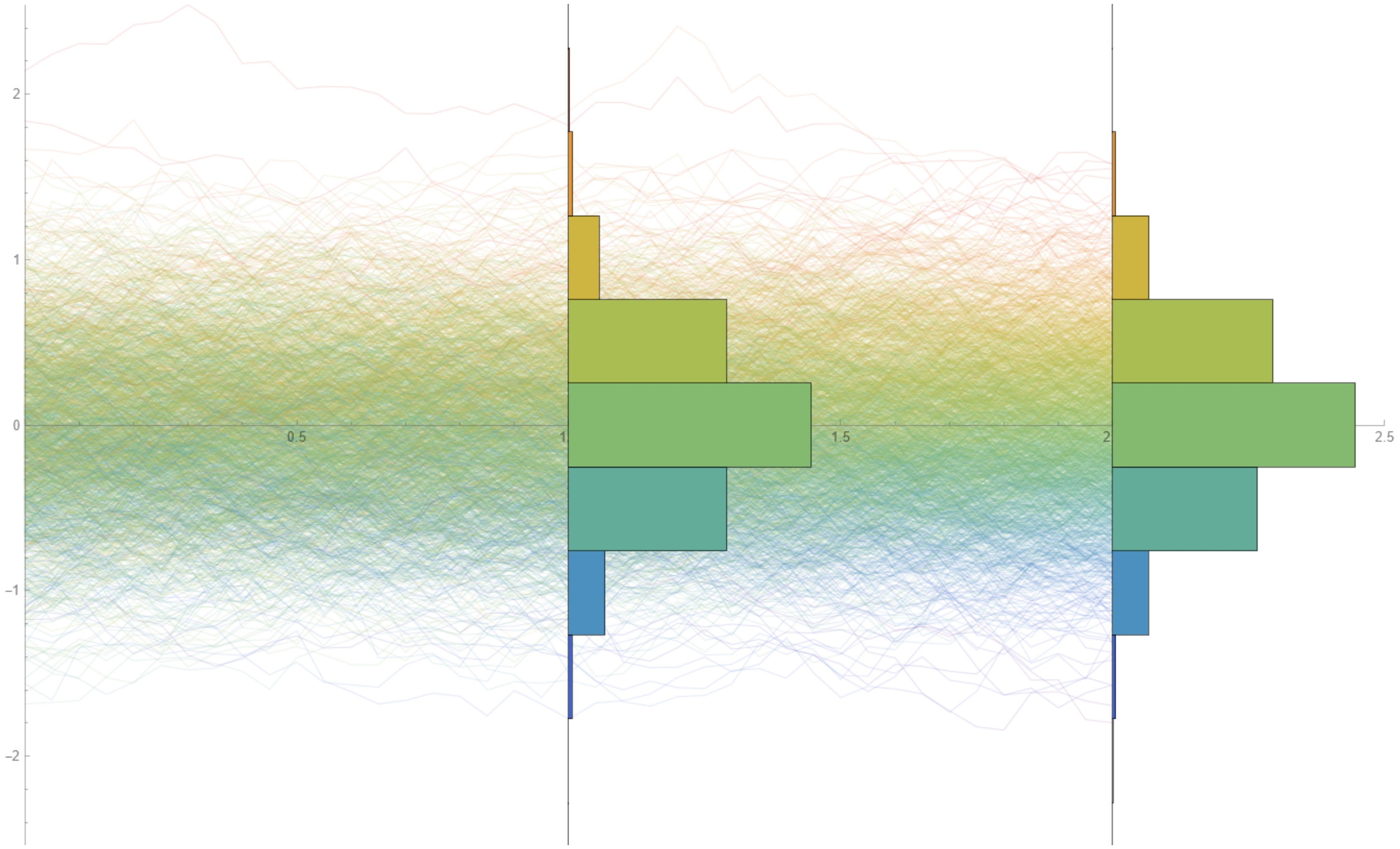

Figure S1. Illustration of an ensemble of realisations of an Ornstein-Uhlenbeck process. Each curve is a different realisation, independent of the others. The process is sliced at two time points $t = 1$ and $t = 2$. Although the individual realisations (curves) take different values at these two time points, they collectively preserve the same Gaussian distribution. This property is known as stationarity and can be assumed when using spectral DCM. Non-stationary processes have different distributions at different time points and would require different models that allow for time varying parameters, e.g. stochastic DCM or adiabatic DCM.

REAL AND IMAGINARY PARTS OF THE CROSS-SPECTRAL DENSITY

The Fourier transform produces complex numbers, which can be described in Cartesian coordinates (real and imaginary parts on the complex plane) or in polar coordinates (amplitude and argument). Polar coordinates offer an intuitive interpretation: the amplitude of the Fourier transform denotes how strongly a given frequency is represented in the signal, while the argument indicates how much the contribution of this frequency is phase-shifted. In Cartesian coordinates, the interpretation is less intuitive: the real and imaginary parts of the cross-spectral density are the Fourier transforms of the even and odd parts of the cross-covariance function. What are the even and odd parts? Before giving the mathematical definition, consider the sine and cosine functions. The cosine function produces the same output regardless of the sign of its input, that is, $\cos(t) = \cos(-t)$. Functions with this property are symmetric with respect to the vertical axis of the Cartesian plane and are referred to as *even*. On the other hand, the sine function flips sign when the input does: $\sin(t) = -\sin(-t)$. Functions with this property are *odd*. Not all functions are purely even or purely odd. However, given a real function $f(t)$, it is always possible to compute its even and odd parts

$$f_{\text{even}}(t) = \frac{f(t) + f(-t)}{2} \tag{S1}$$

$$f_{\text{odd}}(t) = \frac{f(t) - f(-t)}{2} \tag{S2}$$

such that $f(t) = f_{\text{even}}(t) + f_{\text{odd}}(t)$. The cosine function has zero odd part; the sine function has zero even part; and most functions have a mixture of both. This also applies to the cross-covariance function. The Fourier transforms of the even and odd parts of the cross-covariance function produce the real and imaginary parts of the cross-spectral density (Fig. 3 and off-diagonal plots in Fig. 5). A special case of the cross-covariance function is the autocovariance function, which is always even. This is why the diagonal plots in Fig. 5 (the spectral density plots) have zero imaginary part. We refer the interested reader to (Oppenheim et al., 1997) to build further intuition about the Fourier transform and the basics of signal processing.

ANALYTIC SOLUTION FOR THE CORRELATION MATRIX

Integrating the cross-spectral density in Fig. 5 over all frequencies gives us the 3×3 symmetric correlation matrix R, typically used to quantify the functional connectivity. In this example, we can solve the integral analytically to show the explicit dependence of all the pairwise correlation values on the single effective connectivity parameter a_{21}:

$$\rho_{11} = \rho_{22} = \rho_{33} = 1 \tag{S3}$$

$$\rho_{12} = \rho_{21} = \frac{2.7a_{21}}{\sqrt{17.6a_{21}^2 + 10.5}} \tag{S4}$$

$$\rho_{13} = \rho_{31} = \frac{5.9a_{21} - 1.4}{\sqrt{a_{21}\,(137a_{21} - 45.7) + 54.6}} \tag{S5}$$

$$\rho_{23} = \rho_{32} = \frac{a_{21}\,(45.7a_{21} - 8.8) + 13.2}{\sqrt{\left(17.6a_{21}^2 + 10.5\right)\left(a_{21}\,(137a_{21} - 45.7) + 54.6\right)}}. \tag{S6}$$

These functions are plotted in Fig. 7.

DCM DIAGNOSTIC FUNCTION AND VISUAL REPORT

Fig. S2 shows the output of the DCM diagnostics function `spm_dcm_fmri_check()` generated using SPM12. This report complements the cross-spectral density plots in Fig. 6 and additionally indicates the percentage of variance explained (R^2), which is a useful performance metric to judge the quality and success of the model fit to the observed cross-spectral density of the data. The posterior expectation and variance of the effective connectivity parameters are plotted, as well as the posterior correlations among all the model parameters. This figure is just used to illustrate the diagnostic window, not to display the best-case scenario in terms of quality of fit.

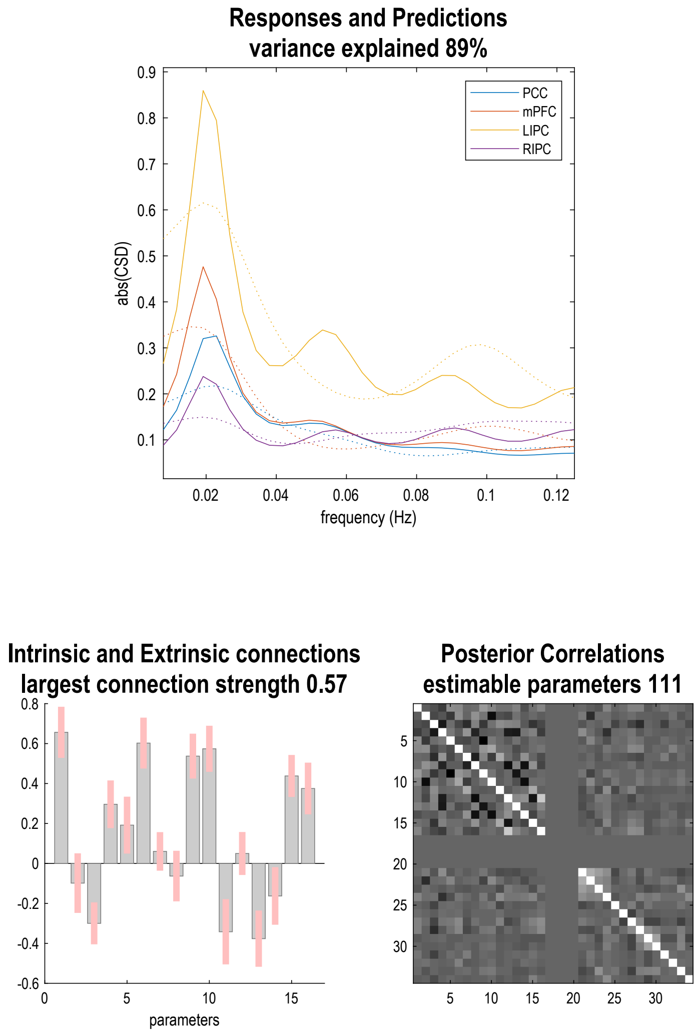

Figure S2. Output of the DCM diagnostics function `spm_dcm_fmri_check()` generated using SPM12. This report indicates the percentage of variance explained (R^2), which is a useful performance metric to judge the quality and success of the model fit to the observed cross-spectral density of the data. Top: BOLD power spectral density obtained via spectral DCM analysis of a real resting-state fMRI dataset described in the *Simulated and empirical cross-spectral density* section. The dashed lines represent the predicted power spectral density and the solid lines the observed ones. Bottom left: Posterior expectation (grey bars) and posterior variance (pink bars) of the effective connectivity values. Bottom right: Posterior correlation matrix of model parameters.
